# Bowel Preparation for Colonoscopy Changes Serum Composition as Detected by Thermal Liquid Biopsy and Fluorescence Spectroscopy

**DOI:** 10.3390/cancers15071952

**Published:** 2023-03-24

**Authors:** Sonia Hermoso-Durán, María José Domper-Arnal, Pilar Roncales, Sonia Vega, Oscar Sanchez-Gracia, Jorge L. Ojeda, Ángel Lanas, Adrian Velazquez-Campoy, Olga Abian

**Affiliations:** 1 Institute of Biocomputation and Physics of Complex Systems (BIFI), Joint Unit GBsC-CSIC-BIFI, University of Zaragoza, 50018 Zaragoza, Spain; 2 Aragón Health Research Institute (IIS Aragón), 50009 Zaragoza, Spain; 3 Centro de Investigación Biomédica en Red de Enfermedades Hepáticas y Digestivas (CIBERehd), 28029 Madrid, Spain; 4 Department of Gastroenterology, Lozano Blesa Clinic University Hospital, 50009 Zaragoza, Spain; 5 Department of Electronic Engineering and Communications, University of Zaragoza, 50009 Zaragoza, Spain; 6 SOTER BioAnalytics, Enrique Val, 50011 Zaragoza, Spain; 7 Department of Statistical Methods, University of Zaragoza, 50009 Zaragoza, Spain; 8 Department of Medicine, Psychiatry and Dermatology, University of Zaragoza, 50009 Zaragoza, Spain; 9 Department of Biochemistry and Molecular and Cell Biology, University of Zaragoza, 50009 Zaragoza, Spain

**Keywords:** thermal liquid biopsy, colonoscopy, colorectal cancer, fluorescence spectroscopy

## Abstract

**Simple Summary:**

Considering thermal liquid biopsy (TLB) as a potential secondary test after fecal immunochemical test positivity (FIT+), the aim of this work was to study possible interferences of colonoscopy bowel preparation on TLB outcome on a retrospective study. TLB parameters together with fluorescence spectra and other serum indicators (albumin and C-reactive protein) confirmed the statistically significant differences between normal colonoscopy patients with and without bowel preparation, revealing the distorting effect of bowel preparation on serum composition and discrimination of disease status. The diagnostic capability of other liquid-biopsy-based methods might also be compromised. Blood extraction after bowel preparation for colonoscopy should be avoided.

**Abstract:**

(1) Background: About 50% of prescribed colonoscopies report no pathological findings. A secondary screening test after fecal immunochemical test positivity (FIT+) would be required. Considering thermal liquid biopsy (TLB) as a potential secondary test, the aim of this work was to study possible interferences of colonoscopy bowel preparation on TLB outcome on a retrospective study; (2) Methods: Three groups were studied: 1/514 FIT(+) patients enrolled in a colorectal screening program (CN and CP with normal and pathological colonoscopy, respectively), with blood samples obtained just before colonoscopy and after bowel preparation; 2/55 patients from the CN group with blood sample redrawn after only standard 8–10 h fasting and no bowel preparation (CNR); and 3/55 blood donors from the biobank considered as a healthy control group; (3) Results: The results showed that from the 514 patients undergoing colonoscopy, 247 had CN and 267 had CP. TLB parameters in these two groups were similar but different from those of the blood donors. The resampled patients (with normal colonoscopy and no bowel preparation) had similar TLB parameters to those of the blood donors. TLB parameters together with fluorescence spectra and other serum indicators (albumin and C-reactive protein) confirmed the statistically significant differences between normal colonoscopy patients with and without bowel preparation; (4) Conclusions: Bowel preparation seemed to alter serum protein levels and altered TLB parameters (different from a healthy subject). The diagnostic capability of other liquid-biopsy-based methods might also be compromised. Blood extraction after bowel preparation for colonoscopy should be avoided.

## 1. Introduction

Although colorectal cancer (CRC) incidence is steadily rising worldwide, its mortality has greatly decreased in recent decades [1]. This reduction is largely attributed to the implementation of population-based screening programs. Screening programs have been shown to reduce CRC mortality in men and women at screening age [1,2]. Colonoscopy is the gold standard technique for CRC detection (statistical sensitivity between 92 and 99%) [3] and it allows, not only the detection of a pathology at an early stage, but also the removal of noncancerous polyps preventing their progression to CRC. However, colonoscopy is an invasive technique, with considerable risk and discomfort for the patient.

Currently, the fecal immunochemical test (FIT) for occult blood testing is the most widely used standard noninvasive method for detecting precursor lesions and early stage CRC prior to colonoscopy [4,5]. Although FIT is cost-effective, it has low clinical potential due to its high false positive rate regarding colonoscopy outcome [6]: about 50% of theFIT(+) colonoscopies performed reveal no findings [7]. A low statistical specificity test not only increases the cost of the screening program but also unnecessarily exposes patients to an invasive and risky procedure such as colonoscopy. In addition, it increases waiting lists for colonoscopy [6] and thus delays the diagnosis of true positives.

TLB analyzes plasma or serum samples using differential scanning calorimetry (DSC). The TLB thermogram reflects the global denaturation of major proteins in serum, which has been shown to be different in healthy and diseased subjects. Other groups have applied DSC to serum/plasma samples associated to inflammation and tumor diseases [8,9,10,11,12]. In our group, we have been working on the development of thermal liquid biopsy (TLB) as a diagnostic technique, applying it to different pathologies such as gastric adenocarcinoma [13], melanoma [14], lung cancer [15], pancreatic cyst [16], and multiple sclerosis [17]. Our goal is to propose an additional test based on TLB for reducing the number of colonoscopy unnecessary arising from FIT(+). Thus, once a case is identified as FIT(+), and prior to prescribing the colonoscopy procedure, TLB would offer the clinician a secondary test helping in the decision to whether or not prescribe a colonoscopy and providing a tool to stratify/prioritize patients in the waiting list. When TLB is applied to a serum sample, a TLB score between zero and one is obtained from the TLB profile, indicating a low (value close to zero) or high (value close to one) probability of presenting a pathology. As any other technique similar to liquid biopsy, TLB is a minimally invasive and low-cost test that could be very useful when applied sequentially after a FIT(+) result. Thus, patients who are FIT(+) and have a TLB score above 0.5 would be further considered for colonoscopy.

Several techniques based on fluid biopsy have been developed in the last decade. It might be reasonable to think that their application in routine clinical practice would be straightforward, but there are still some practical issues that need to be addressed before their extensive use in the clinic [18]. Some concerns are related to biomarker levels/thresholds in samples, a lack of consensus on the preanalytical and analytical conditions, clinical validation, regulatory compliance, and cost-effectiveness [19,20,21]. These methodological problems have led to the current situation where the use of fluid biopsy in clinical practice is limited [21,22,23]. Therefore, it is necessary to work towards a consensus that will allow a full implementation of fluid biopsy techniques in the near future. The advantages of the technique are obvious: information-rich, minimally invasive, low cost, and high adherence for periodic testing. In the case of TLB, an aspect needs to be addressed: the preanalytical conditions during the sample extraction.

Many validation studies for liquid biopsy applied to serum diagnosis of CRC are usually carried out with patient samples collected at the same time as the colonoscopy procedure to avoid an additional hospital visit [24,25,26,27,28]. Standard preparation for colonoscopy includes two days on a low-residue diet with 24 h of fasting (fluid intake only) and administration of laxatives prior to the procedure. There are studies reporting altered patient serum composition after preparation for colonoscopy, and therefore, any analysis (e.g., proteomic, metabolomic) performed on those samples may potentially be distorted [24,25,26,27,28].

In this study, we examined how patient preparation associated with the colonoscopy procedure affects the TLB outcome. Additionally, we provided further evidence for changes in protein serum composition using serum fluorescence spectroscopy and concentrations of albumin and C-reactive protein (CRP) measured by conventional biochemical methods.

## 2. Materials and Methods

### 2.1. Subjects and Samples

#### 2.1.1. Serum Samples for Primary TLB Study

The first group of serum samples (n = 514) was collected from patients enrolled in the regional CRC screening program at Hospital Clínico Universitario Lozano Blesa (HCULB) in Zaragoza, from April to October of 2019, and FIT was positive in all subjects (cut-off value of 20 µg hemoglobin per gram of stool). Samples were drawn the same day of the colonoscopy procedure, when patients were under standard bowel preparation (Citrafleet^®^). Clinical variables were collected from the patients, such as demographic data (age and sex) and colonoscopy diagnosis (gastroenterologist’s findings confirmed by a histopathological analysis).

The first group of patients was classified into: 1. Normal group (CN, n = 247), including subjects with non-neoplastic colon pathologies for whom the FIT(+) result could be justified with other pathologies such as hemorrhoids, diverticulitis, fissures, or nonpathological (hyperplastic) polyps. This group also included subjects with no findings in their colonoscopies, but with a FIT(+) diagnosis. 2. Pathological group (CP, n = 267), including subjects with any of the following colonoscopy findings: 2.A. nonadvanced polyp (tubular adenoma <10 mm and with a low-grade dysplasia or serrated polyp <10 mm and without dysplasia); 2.B. advanced polyp (adenoma with villous histology, ≥10 mm, or with high-grade dysplasia; or serrated polyp ≥10 mm or with dysplasia; or more than two lesions (adenomas or serrated polyps)), independently of the characteristics of the lesions; and 2.C. CRC.

Symptomatic patients with a prescribed colonoscopy were excluded. Subjects with a history of inflammatory bowel disease, elevated risk of CRC (familial CRC, hereditary CRC, and polyposis syndromes, including Lynch Syndrome, familial adenomatous polyposis, MUTYH-associated polyposis, serrated polyposis syndrome, and hamartomatous polyposis condition, such as Peutz–Jeghers syndrome and juvenile polyposis syndrome), and subjects who underwent colonoscopy referred for polyps and/or under CRC surveillance were also excluded. Other exclusion criteria were patients with an incomplete colonoscopy in which the cecum was not reached, with a mean withdrawal time of less than 6 min, or with inadequate bowel preparation according to the validated Boston Bowel Preparation Scale. Therefore, asymptomatic patients with complete visualization of the colon and appropriate withdrawal time were included, as well as those with an incomplete colonoscopy due to a neoplastic structure.

A summary of the characteristic data of these patients, is provided in Table S1.

#### 2.1.2. Serum Samples for Secondary TLB Study

Given potential serum alterations as a consequence of the bowel preparation for a colonoscopy, a second group of samples was collected: a subcohort of 55 patients already enrolled in the primary TLB study (from CN) with normal colonoscopy diagnosis (no pathological findings) were invited to participate in a secondary study by resampling (CNR, n = 55). These patients were requested, from January to February 2020, eight months (on average) after their colonoscopy procedure, to attend a blood draw appointment under standard preanalytical conditions, corresponding to a routine blood test (about 8–10 h fasting without any other treatment).

The third group of patients was a cohort of 55 healthy blood donors (similar in age and sex to the 55-subject CNR subcohort) from a regional blood bank (BD, n = 55) who were considered as a healthy control. Their serum samples were drawn under the same standard preanalytical conditions (about 8–10 h fasting) and they had no evidence of disease (blood bank ensured the quality of these samples through a health questionnaire and a standard blood transmission pathogen analysis).

**Figure 1:**
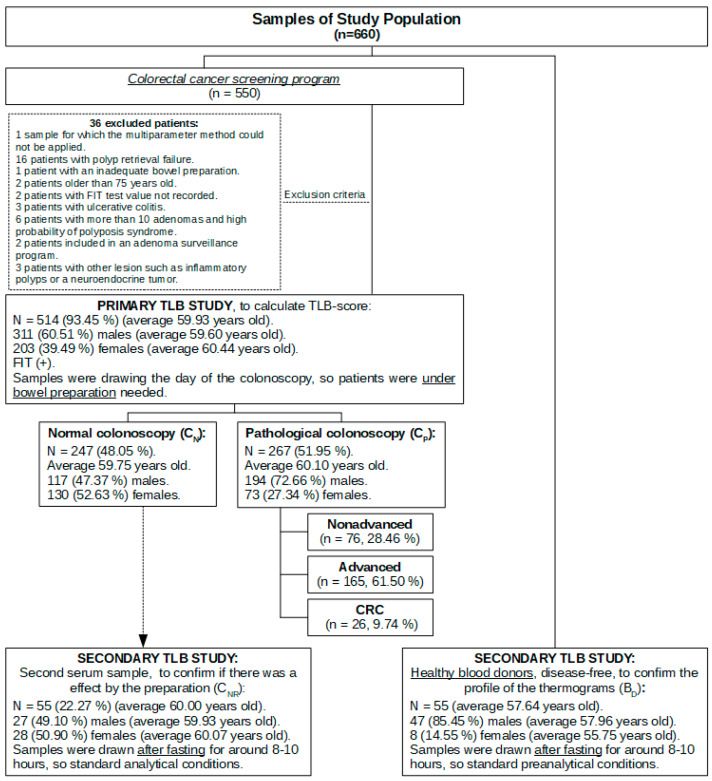
Sample study population flow chart.

An overview of the design and detailed summary of the subjects recruited in these primary and secondary studies is shown in Figure 1.

All participating volunteers were informed of the purpose and nature of the study before giving written consent. The study protocol was approved by the Regional Research Ethics Committee in Zaragoza (Spain, CI: PI19/059 dated 13 March 2019, no. 05/2019). The preanalytical conditions of all the participants by the time of the sample extraction were registered in the sample data file.

### 2.2. Blood Samples Processing

Five milliliters of blood were drawn and collected in tubes with separating gel (BD Vacutainer^®^), after which they were centrifuged at 3000 rpm for 10 min and immediately aliquoted and stored at −80 °C. All samples were labeled with an internal code to guarantee patient anonymity following the protocol described in the procedure approved by the ethics committee.

### 2.3. TLB Profile Determination

TLB profiles and TLB scores were determined after analyzing the thermograms obtained from the serum samples. These thermograms were recorded using an automated high-sensitivity differential scanning VP-DSC microcalorimeter (MicroCal, Malvern-Panalytical, Malvern, UK). The thermogram consists of the experimental determination of the excess heat capacity of the sample, with regard to a buffer reference solution, as a function of temperature along the thermal denaturation process. Each serum sample was diluted (1:25) in filtered phosphate-buffered saline (PBS) and 400 μL was taken for the assay. Experiments were performed at a scanning rate of 1 °C/min. Thermograms were baseline-corrected and analyzed using software developed in our laboratory implemented in Origin 7 (OriginLab).

### 2.4. Fluorescence Spectroscopy

From each serum sample diluted (1:25) in PBS, 80 μL was transferred into 96-well microplates (96-well PCR plate, non-skirted, from 4titude, Waltham, MA, USA). Fluorescence measurements were performed in a CLARIOstar plate reader (BMG Labtech, Ortenberg, Germany) by selecting an excitation wavelength of 330 nm, with excitation and emission bandwidths of 10 nm, and recording the fluorescence emission spectra in the 400–700 nm range.

From the fluorescence spectra, F(λ), where F is the fluorescence emission intensity at wavelength λ, two parameters (Max and SNR) were calculated. Max is the maximal fluorescence intensity of the spectrum, whereas the SNR (signal-to-noise-ratio) quantifies the fluorescence signal relative to the sample noise.

### 2.5. Albumin and C-Reactive Protein Serum Concentration

Albumin concentration was determined by employing a Flex^®^ reagent cartridge (Dimension^®^ clinical chemistry system, Siemens, Erlangen, Germany), which is an adaptation of the bromocresol purple (BCP)-dye-binding method reported by Carter [29]. Because of an enhanced specificity of BCP for albumin, this method is not affected by globulin interference [30]. Multiple wavelengths blanking increases the sensitivity and minimizes the spectral interference from lipemia. The reference values for albumin were 3.2–4.8 g/dL.

The CRP concentration was determined with the Dimension^®^ clinical chemistry system, based on a particle-enhanced turbidimetric immunoassay (PETIA) technique. The reference values for the CRP were 0–5.0 mg/L.

### 2.6. Data and Statistical Analysis

TLB thermogram raw data were processed using Origin software (OriginLab, Northampton, MA, USA) as previously described [13]. We have developed a multiparametric phenomenological model in which the TLB serum thermogram is deconvoluted into six individual transitions, modeling each individual transition using the logistic peak or Hubbert function [13,15]. This model has been successfully applied in the analysis of serum samples from patients with melanoma, gastric and lung cancer, pancreatic cysts, and multiple sclerosis [13,14,15,16,17]. The primary parameters obtained directly from the thermogram deconvolution (center temperature, height, and width of each individual transition) were combined to define a new final set of 11 parameters, more convenient for extracting and summarizing the essential geometric features of the thermogram. The parameters obtained in this multiparametric data analysis were used to derive the TLB score by applying a generalized linear model (GLM) [15]. The TLB score represents the probability of an individual to show serum alterations according to the corresponding TLB thermogram. Therefore, the TLB score is a single value for classifying a given subject as healthy or diseased, with a certain probability, as it was previously proven for lung cancer and multiple sclerosis [15,17]. Like any probability, the TLB score ranges from 0 to 1: the model classifies the subjects as exhibiting serum alterations (i.e., expected pathological outcome at colonoscopy) for TLB score values >0.5, and lacking relevant serum alterations (i.e., expected normal outcome at colonoscopy) for values <0.5. The performance of the diagnostic test was evaluated by calculating common performance indexes (sensitivity, specificity, positive predictive value, and negative predictive value), as well as the receiver operating characteristic (ROC) curve.

The Kolmogorov–Smirnov–Lilliefors test or the Shapiro–Wilk test (according to sample size) was performed to assess the normality of the variables. Averages between two independent/dependent groups were compared using the Student *t*-test for variables with normal behavior (previously verifying homoscedasticity with Bartlett’s test), whereas medians between two independent/dependent groups were compared using the Wilcoxon test for variables with a non-normal behavior. The dependence between groups for dichotomous qualitative variables was assessed using the Pearson chi-square test, and the dependence between a dichotomous group and an ordinal group (three or more categories) was assessed using a linear Pearson chi-square test. Medians between three or more independent groups were compared using the Kruskal–Wallis test (previously verifying homoscedasticity with Levene’s test) for variables with a non-normal behavior.

The statistical analyses were performed using Rstudio, R version 3.6.1 (5 July 2019). For all tests, a two-sided *p*-value of less than 0.05 was considered statistically significant. In case of multiple testing, *p*-values were adjusted according to the FDR method.

## 3. Results

### 3.1. Clinical Characteristics of the Study Population

In the first group, we recruited a total of 550 FIT(+) patient samples drawn for this study. Of these, 514 (93.45%) were analyzed and 36 were discarded due to different methodological reasons: 16 patients with polyp retrieval failure, 6 patients in whom more than 10 adenomas were found (indicating a high probability of polyposis syndrome), 3 patients with ulcerative colitis, 3 patients with other lesion such as inflammatory polyps or neuroendocrine tumor, 2 patients older than 75 years, 2 patients included in an adenoma surveillance program, 2 patients with a FIT value not recorded, 1 patient with inadequate bowel preparation, and 1 patient whose sample could not be accurately analyzed by DSC for applying TLB.

According to sex, 311 and 203 samples (60.51% and 39.49%) corresponded to males and females, respectively. According to age, there were no statistically significant differences: A. in median age between sexes (*p*-value = 0.091, Wilcoxon test for independent samples); B. in median age between normal colonoscopy or pathological diagnosis (*p*-value = 0.427, Wilcoxon test for independent samples); and C. in median age between each colonoscopy result (*p*-value = 0.275, Kruskal–Wallis test for independent samples). A complete and detailed descriptive analysis of age and sex can be found in the Supplementary Materials section (Table S2).

A certain association was found between sex and pathological colonoscopy diagnosis (*p*-value < 0.001, Pearson chi-square test; Table S3). In addition, the greater the severity of the pathology, the higher the percentage of males compared to the female patient cohort (*p*-value < 0.001, linear Pearson chi-square test) (Figure S1A, Table S4). Colonoscopy diagnosis revealed that almost half of them (247/514, 48.05%) were classified as normal (FIT (+) with no pathological findings, CN). Moreover, for those with colonoscopy findings (CP), they were classified into several diagnostic groups: 32.10% with an advanced polyp (165/514), 14.79% with a nonadvanced polyp (76/514), and 5.06% with CRC (26/514) (Figure S1B, Table S4).

In the second group of samples (resampling of a subcohort of 55 patients already enrolled in the primary TLB study who showed normal colonoscopy diagnosis, CNR) there was no statistically significant difference between the average age in both sexes (*p*-value = 0.902, Student’s *t*-test for dependent samples; Table S5).

In the third group, a cohort of healthy BD who were considered as healthy controls, there was no statistically significant difference between the median age in both sexes (*p*-value = 0.320, Wilcoxon test for independent samples; Table S6).

### 3.2. Primary TLB Study: Normal Colonoscopy Group vs. Pathological Colonoscopy Group

**Figure 2:**
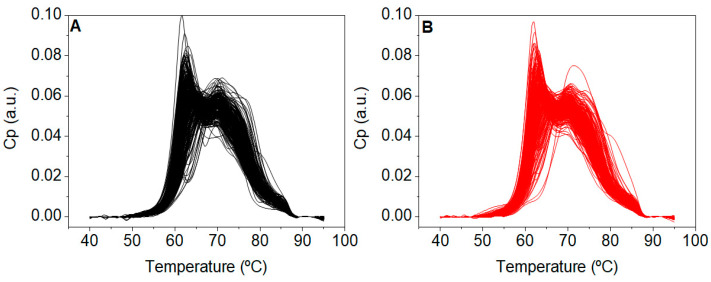
Thermograms from patients with (**A**) normal (CN, n = 247) and (**B**) pathological (CP, n = 267) colonoscopy diagnosis.

TLB thermograms were recorded from all subjects included in the primary study CN and CP (Figure 2). These thermograms reflected serum protein composition and their overall stability against thermal denaturation. Unexpectedly, thermograms from patients with a CN diagnosis were visually very similar to those thermograms from patients with a pathological colonoscopy CP diagnosis.

Each thermogram was analyzed by applying the six-component phenomenological multiparametric deconvolution procedure (as described in [13]), and a final set of eleven TLB-associated parameters was obtained. We determined the three boundaries (Q1, Q2, Q3) of the quartiles, and the minimum and maximum for the distribution of these TLB-associated parameter in each group (Table S7).

A bivariate statistical analysis of each TLB-associated parameter (CN values vs. CP values) was performed in order to identify statistically significant differences according to the colonoscopy diagnosis (normal vs. pathological). Previously, a normality test was performed for each parameter, and therefore, the Wilcoxon test for independent samples was applied (TLB-associated parameters were not normally distributed). None of the parameters showed statistically significant differences between the two groups (patients with CN and patients with CP) with a *p*-value lower than 0.05 being considered significant (Figure S2, Table S8), confirming the initial visual inspection of the thermograms. This result suggested that, unlike previous studies on other tumor diseases (melanoma, lung, and gastric cancer), TLB would not be able to discriminate between CN healthy and CP patients.

A more in-depth study was performed by building a global classification model according to the TLB score value for each subject, following our previously developed methodology [15,17]. Briefly, TLB scores were obtained by applying a multivariate GLM as a statistical classification/predictive tool. TLB scores did not exhibit any statistical significance (all *p*-values >0.05, Table S9) for discriminating between a CN and CP diagnosis. Considering a TLB-score threshold value of 0.5, values between 0 and 0.5 would be considered as a nonpathological status and values between 0.5 and 1 would be considered as a pathological status. The results obtained showed that there were 183 patients with normal colonoscopy exhibiting a TLB score >0.5 (74.1% false positive rate) and 39 patients with a pathological colonoscopy exhibiting a TLB score <0.5 (14.6% false negative rate) (Table S10). In terms of predictive power, the area under the ROC curve value was 0.57 (0.52, 0.61) (Figure S3), revealing a success ratio of 57%, with 85.4% sensitivity and 25.9% specificity. This result pointed to a lack of discriminatory ability of TLB with respect to normal/pathological colonoscopy, derived mainly from an unexpectedly low statistical specificity (most subjects with a normal colonoscopy were classified as diseased subjects).

This result contrasted with previously published studies, in which we proved that GLM applied to TLB was capable of successfully classifying patients into normal and pathological groups, providing a unique value with potential direct application in clinical practice.

**Figure 3:**
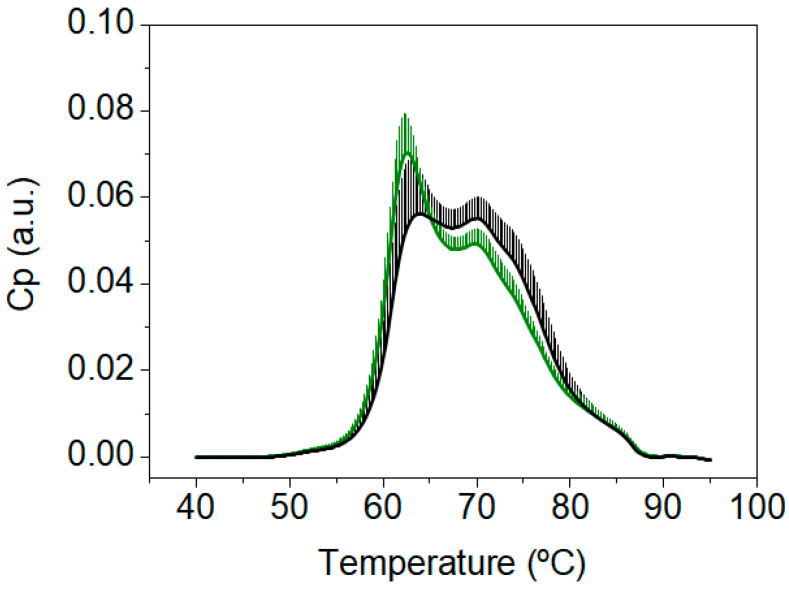
Average and standard deviation of the thermograms from healthy blood donors (BD, green, n = 55), and from patients with a normal colonoscopy diagnosis (CN, black, n = 247).

At this point we wondered: (1) why did the TLB thermograms of patients with a CN and CP diagnosis showed a high similarity? (2) Why was the TLB score unable to successfully classify patients into those groups? (3) Was this drawback the result of a fundamental flaw in TLB specific for CRC? Then, we focused our attention on those subjects with no colonoscopy findings (normal colonoscopy diagnosis, CN) and compared them to the group of healthy blood donors (disease-free subjects, BD). Visually, the TLB thermograms of patients with a CN diagnosis were very different from the TLB thermograms of the group of healthy BD (Figure 3). Moreover, importantly, the TLB thermograms of our healthy BD group in this study were very similar to the TLB profiles from healthy subjects previously reported in the literature by other research groups [31] and in our own work [15].

**Figure 4:**
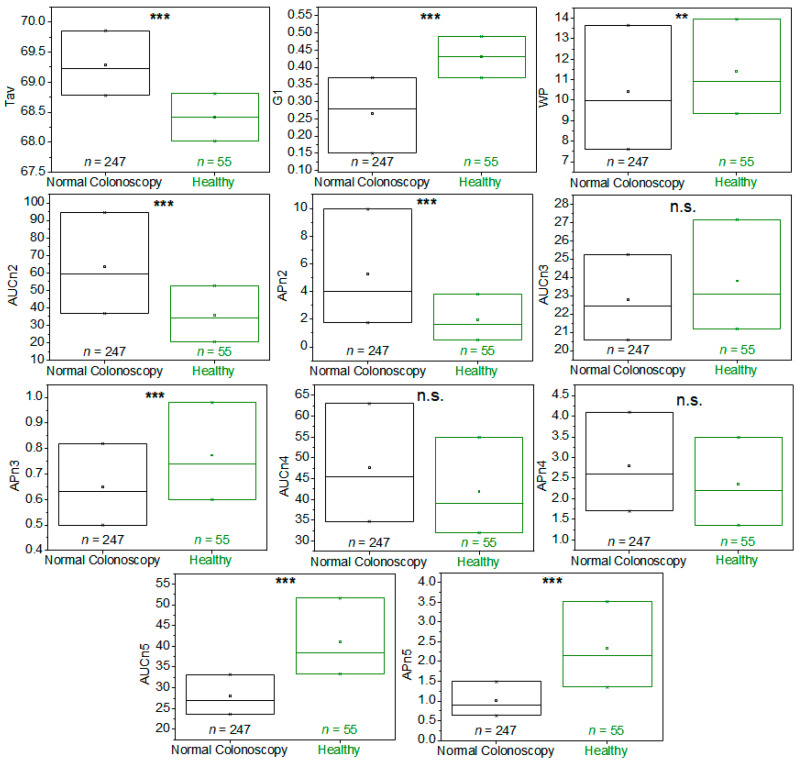
Box-plot diagrams indicating Q1, Q2, and Q3, together with the average value (square) for each TLB parameter, illustrating the distribution of the *p*-values (asterisks indicate the order of magnitude of *p*-values) between patients with a normal colonoscopy diagnosis (CN, black) and healthy blood donors (BD, green). It was calculated according to Student’s *t*-test or a Wilcoxon test for independent samples, depending on the normality character of the parameter distribution. Note: n.s.: *p*-value is not significant; **: *p*-value = 0.050–0.010; ***: *p*-value = 0.009–0.001.

When the TLB parameters were determined for this healthy BD group (detailed description of can be found in Table S11) and the bivariate statistical analysis was performed, statistically significant differences could be determined for all parameters, except AUCn3, AUCn4, and APn4, when comparing patients from the colorectal screening program with a CN diagnosis and healthy BD (*p*-value < 0.05) (Figure 4 and Table S12).

Differences observed in TLB-associated parameters between the CN patients and healthy BD confirmed the features of the thermograms observed visually in Figure 3. This suggested that the subjects from the CN group did not show TLB thermograms typical of healthy BD subjects but showed TLB thermograms similar to those from the CP group. Of course, that would be possible, because the patients undergoing colonoscopy, albeit with no clinical findings, were FIT(+). However, that was a major drawback, because we were interested in discriminating between CN and CP subjects, with the ultimate goal of reducing unnecessary colonoscopy interventions. Then, under the conditions of the study, the CN group was not a good control group for the CP group.

### 3.3. Secondary TLB Study: Resampled Normal Colonoscopy Group vs. Healthy Group

Next, we focused on one of the main differences between CN patients and healthy BD: the preanalytical conditions for blood collection (i.e., colonoscopy bowel preparation vs. standard 8–10 h fasting). We hypothesized that, most likely, the TLB thermograms from the CN patients were altered as a consequence of the harsh conditions imposed by laxative treatment and stringent diet intake, resulting in TLB thermograms similar to those from CP patients. Our direct approach was (1) selecting randomly a subcohort of 55 patients (CNR) from the 247 patients with a CN diagnosis; (2) obtaining new serum samples after standard fasting conditions to be tested by TLB; and (3) comparing the new TLB thermograms with the previous TLB thermograms obtained with the samples collected after colonoscopy preparation.

**Figure 5:**
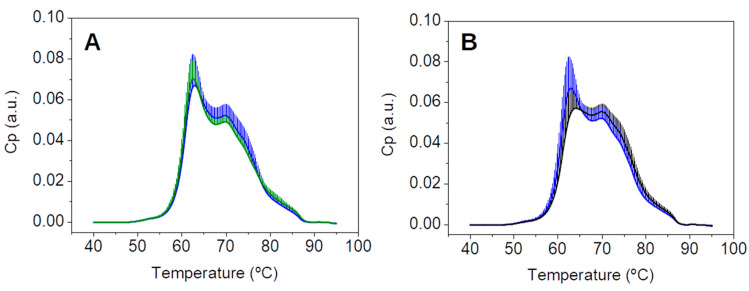
(**A**) Average and standard deviation of the thermograms from healthy BD (green, n = 55) and patients (blue, n = 55); (**B**) average and standard deviation of the thermograms from 55 selected patients with a normal colonoscopy diagnosis in two blood draw conditions: with colonoscopy preparation (CN, black) and with standard analytical conditions (CNR, blue).

When comparing the new TLB thermograms for these 55 patients CNR under standard preparation (CNR) with the TLB thermograms of healthy BD, only minor differences were found (Figure 5A). These results were remarkably different from the results previously described in Figure 3, confirming that these new samples were similar to the well-known healthy TLB profiles. TLB parameters were also determined for these 55 subjects with a CNR diagnosis under standard preanalytical conditions and the detailed description is presented in Table S13.

The direct comparison of average TLB thermograms of the 55 selected patients with a normal colonoscopy under the two different conditions, a first blood sample drawn under a colonoscopy preparation and a second blood sample drawn under standard pre-analytical conditions, is shown in Figure 5B. As it can be observed, the average thermograms exhibited markedly different features. Detailed examples of each patient comparison, corresponding to 16 individual TLB thermograms, are presented in Figure S4. In most of them, the second blood sample (drawn under standard preanalytical conditions) could be easily identified as it showed uniform, conserved features among all patients (blue curves in Figure S4) and, very importantly, they seemed quite similar to the typical healthy TLB thermogram. Regarding the first blood sample (drawn under colonoscopy preparation conditions), a greater variability was found between patients (black curves in Figure S4), and their TLB profiles differed much more from the typical healthy TLB thermogram.

**Figure 6:**
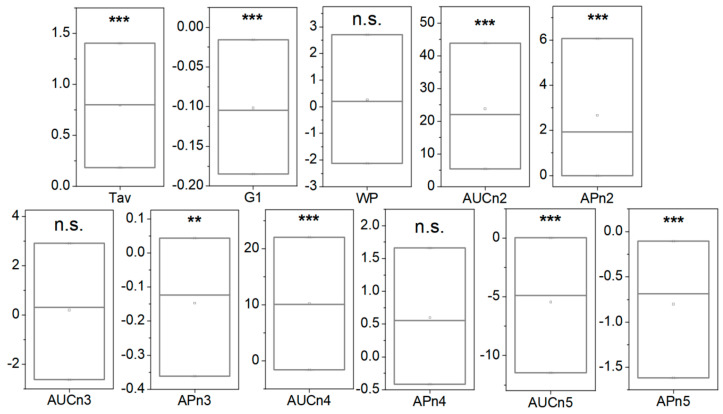
Box-plot diagrams indicating Q1, Q2, and Q3, together with the average value (square) for each TLB parameter, illustrating the difference between 55 samples without preparation and 55 samples with preparation (asterisks indicate the order of magnitude of the *p*-value). Note: n.s.: *p*-value is not significant; **: *p*-value = 0.050–0.010; ***: *p*-value = 0.009–0.001. *p*-values were calculated according to Student’s *t*-test or a Wilcoxon test, both for dependent samples, depending on the normality character of the parameter distribution.

Following our established procedure, the TLB parameters were determined for the second samples of the 55 CNR patients, and a bivariate statistical analysis comparison with the first sample extracted was performed (a detailed description of the 55 patients with preparation can be found in Table S14). Many of the TLB-associated parameters (Tav, G1, AUCn2, APn2, APn3, AUCn4, AUCn5, and APn5) showed statistically significant differences between the averages/medians of both groups (*p*-value < 0.05; *p*-values were calculated according to Student’s *t*-test or a Wilcoxon test, both for dependent samples, depending on the normality character of the parameter distribution) (Figure 6 and Table S15).

Considering these results, at this point it was reasonable to think that TLB profiles from serum samples collected during the colonoscopy procedure might have been distorted by alterations in serum composition as a consequence of the harsh bowel preparation procedure. Of course, this observation could be specific to TLB, since TLB monitors the thermal denaturation behavior of the major proteins in serum. Nevertheless, colonoscopy preparation might also alter other serum components (e.g., circulating cells, miRNA, cfDNA, ctDNA, etc.).

In addition, could this serum alteration upon colonoscopy preparation be detected using other biophysical techniques? Fluorescence has proven to be a versatile tool for studying molecular properties in analytical chemistry, biochemistry, cell biology, photochemistry, and environmental science. Fluorescence detection has four major advantages over other light-based investigation methods: a high sensitivity, high speed, broad availability, and safety. When excited at suitable wavelengths, some biomolecules emit radiation over a wide spectral range in the ultraviolet–visible region. Changes in serum composition will be reflected in changes occurring in biochemical, physicochemical, and histological properties, and in particular, changes in the fluorescence emission spectrum [32].

We analyzed the fluorescence spectrum of the serum samples drawn from the 55 CN and CNR patients with a normal colonoscopy under a colonoscopy preparation and under standard preanalytical conditions (8–10 h fasting). As an example, the fluorescence spectra of 16 subjects (same individuals as in Figure S4) are represented in Figure S5.

The differences in serum fluorescence obtained under the two conditions (with or without colonoscopy preparation) could be easily observed in most cases. However, to compare both fluorescence spectra quantitatively (Figure S5), two parameters from the spectra, the Max and SNR, were calculated as previously described in the Materials and Methods section. The bivariate analysis of the Max and SNR parameters from samples with or without preparation showed statistically significant differences between both groups (*p*-value < 0.05; *p*-values were calculated according to Student’s *t*-test for dependent samples) (Figure S6 and Table S16).

Finally, looking for additional evidence for alterations caused by the bowel preparation, we determined the concentration of two serum proteins used in clinical diagnostic tests: albumin (main serum protein) and CRP (acute phase protein) concentrations, both in the 55 normal colonoscopy patients under colonoscopy preparation and under preanalytical conditions. As expected, there were statistically significantly differences (*p*-value < 0.05; *p*-values were calculated according to Student’s *t*-test for dependent samples) in serum albumin and CRP concentration levels between CN samples and CNR samples (Figure S7, Table S17).

## 4. Discussion

Current CRC diagnosis in cancer screening programs is performed through a combination of FIT and colonoscopy when required. FIT is convenient and appropriate for patient compliance, but it suffers from a high false positive rate. On the other hand, colonoscopy provides direct evidence of malignancy but presents a potential risk of serious complications for the patient, is a resource-intensive procedure, and is characterized by a notable discomfort for the patient. Therefore, new efficient, simple, inexpensive diagnostic tools are needed for clinical practice to improve patient management and increase quality of life during surveillance and monitoring.

Towards this goal, we proposed a study to develop a simple, risk-free, and low-cost clinical test to discriminate FIT(+) patients with a high risk of CRC who should undergo a further colonoscopy procedure, with the aim of reducing the excessive number of unnecessary colonoscopies in current screening programs. Because thermal liquid biopsy (TLB) measures the denaturation profile of blood samples in a simple manner and it is very sensitive to changes in serum protein composition associated with cancer [13,14,15], we considered it a suitable technique for this purpose.

We compared the TLB thermograms of 247 patients from a colorectal screening program with normal colonoscopy (CN) with the TLB thermograms of 267 patients with pathological colonoscopy (CP), all subjects being FIT(+). Samples were obtained the same day of the colonoscopy, and patients were under bowel preparation. The TLB thermograms from CN subjects were similar to those from CP subjects, resulting in a TLB score with a low predictive ability (AUC ROC of 0.57 (0.52, 0.61)). After these results and a thorough review of the study process, we put into question the sample extraction procedure itself (timing, patient preparation, mainly). First, thermograms from the CN (normal colonoscopy result) group were compared with an additional group of 55 age- and sex-matched healthy blood donors (BD), whose thermograms matched those previously reported in the literature for healthy subjects [31]. We observed significant differences between CN and BD thermograms, as well as in the TLB parameters derived from them. All these results indicated CN was not an appropriate control group for CP and for developing a good classification model. Next, we collected a second serum sample from 55 patients from the CN group, but this time blood samples were drawn under standard pre-analytical conditions (8–10 h fasting, CNR) and no bowel preparation eight months after colonoscopy. Here, it is important to note that previous studies in our group showed that the TLB profile for a given subject under the same preanalytical conditions remained unaltered with time, unless a pathological event arose; annual samples in the absence of diagnosed disease were compared and TLB was almost identical after a six-year follow-up. Therefore, TLB profiles are very robust over time and not affected much by additional factors other than disease. The TLB thermograms from the CNR group (standard preanalytical conditions) were significantly different from those of the CN group (after bowel preparation for colonoscopy) and were very similar to those from the (BD) healthy blood donor group, indicating that the CNR group was an appropriate control group for CRC screening purposes. In this comparison, we selected the same patients, with and without bowel preparation (paired samples), in order to reduce interindividual variability in the comparison.

Summarizing the results: (1) bowel preparation for colonoscopy alters the composition of serum to a large extent, and consequently, the TLB thermograms of patients with a normal colonoscopy (CN) are similar to those of patients with pathological findings (CP); (2) TLB thermograms of samples collected after standard preanalytical conditions (8–10 h fasting) from patients with a normal colonoscopy are similar to TLB thermograms from healthy blood donor subjects (BD); and (3) TLB thermograms from samples collected after standard preanalytical conditions (8–10 h fasting) from patients with a normal colonoscopy (CNR) are different from TLB thermograms from samples drawn from the same subjects drawn under bowel preparation conditions (CN). Thus, preanalytical conditions are relevant for the reliability of the analytical test based on TLB. Bowel colonoscopy preparation alters blood protein (and possibly other components) levels, rendering TLB (and possible other clinical tests based on serum composition) unreliable in terms of classifying patients according to colonoscopy outcomes. These results reveal that the timing and conditions for blood draw (or, in other words, the preanalytical conditions of the patient) are a key factor for obtaining meaningful data for diagnosis or surveillance from blood samples.

As the TLB thermogram reveals the behavior of the major serum proteins against thermal denaturation, bowel preparation must alter the serum protein composition. In particular, we assessed the alteration of protein concentration levels in serum by fluorescence spectroscopy and by determining the concentration of individual proteins in serum: albumin (major serum protein) and C-reactive protein (acute phase protein). In all cases, there were statistically significant differences between the two groups (same patient with and without bowel preparation).

In the clinical routine and research protocols conducted in CRC screening programs, it is usual to obtain blood samples at the time of the colonoscopy procedure, but this is something that should be reconsidered. The results reported in this manuscript would represent a call of attention for past, current, and future studies with this type of design. Certainly, the results reported here will contribute to establish optimal preanalytical conditions for TLB.

As in any other analytical technique, there are some limitations in our study based on TLB. TLB is a technique reporting an overview of the protein composition of plasma/serum samples, reflecting changes in protein concentrations, and/or changes in structural stability elicited by interactions with disease-specific biomolecules. Thus, it is not possible, at this moment, to discriminate the molecular basis for the alterations observed in the thermograms of diseased patients. TLB provides a phenomenological observation reflecting alterations caused by colorectal cancer, and this study was prompted by the necessity to remove other (non-disease-related) sources of changes in plasma composition.

## 5. Conclusions

The study presented in this work clearly demonstrated that preanalytical conditions must be well-established and defined for diagnostic techniques based on blood samples, such as TLB. We propose that, although logistically convenient, blood collection should be avoided when the patient is under colonoscopy preparation conditions; otherwise, considerable and significant changes in serum composition may distort the analytical outcome of the test on the fluid biopsy sample. It is essential to establish robust preanalytical conditions to ensure the accuracy and the reliability of the test. In our case, the TLB study validation in colorectal screening program has been currently redesigned according to these conclusions and, since then, patient serum samples have been collected under standard preanalytical conditions (8–10 h fasting). A manuscript with the results will be published soon.

It is important to emphasize that alterations observed in TLB and caused by the preanalytical conditions suggest there is a change in plasma/serum composition, and this could also affect and alter the outcome from other analytical clinical tests based on quantifying other biomarkers (proteins and nucleic acids).

## Data Availability

The data presented in this study are available on request from the corresponding author.

## Supplementary Materials

The following supporting information can be downloaded at: https://www.mdpi.com/article/10.3390/cancers15071952/s1, Figure S1: (A) Proportion of males (dark grey) and females (light grey) according to the colonoscopy result, for patients from CRC screening program. (B) Distribution of colonoscopy results for patients from CRC screening program. Figure S2: Box-plot for each individual TLB-parameter illustrating the distribution of the *p*-values between patients with normal (CN) or pathological colonoscopy (CP) diagnosis. Figure S3: ROC curve illustrating the statistical performance of the TLB-score and its AUC (95 % CI) for discriminating patients with CN diagnosis from patients with CP diagnosis. Figure S4: Comparison of individual examples of 16 patient thermograms with normal colonoscopy diagnosis. Samples were drawn in two different conditions: under colonoscopy preparation (CN, black) and under standard analytical conditions (CNR, blue). Figure S5: Comparison of individual examples of 16 patient fluorescence spectra with normal colonoscopy diagnosis. Samples were drawn in two different conditions: under colonoscopy preparation (CN, black) and under standard pre-analytical conditions (CNR, blue). Figure S6: Plot and distribution of the difference in MAX and SNR between samples without preparation and samples with preparation. Figure S7: Plot and distribution of the difference in albumin concentration and C-reactive protein concentration between samples without preparation and samples with preparation. Table S1. Summary of characteristics data of patients from colorectal cancer (CRC) screening program, with fecal immunochemical test positive. Table S2. Age distribution for patients from (CRC) screening program according to the sex and to the colonoscopy findings. Table S3. Descriptive of sex for patients from (CRC) screening program depending on colonoscopy result. Table S4. Descriptive of sex for patients from (CRC) screening program depending on each category of colonoscopy result. Table S5. Age distribution for patients from (CRC) screening program with normal colonoscopy result and without colonoscopy preparation (CNR, n = 55) according to the gender. Table S6. Age distribution for healthy blood donors (BD, n = 55) according to the sex. Table S7: Main descriptive indexes for the eleven TLB-associated parameters in patients with normal colonoscopy result (CN, n = 247) and pathological colonoscopy result (CP, n = 267). Table S8: Bivariate analysis of TLB parameters of patients with normal colonoscopy result (CN) and patients with pathological colonoscopy result (CP). Table S9: Summary of the application of multivariate Binomial Generalized Linear Model with Logistic Regression (GLM). Table S10: Contingency table for predict risk of pathological colonoscopy finding. Table S11: Main descriptive indexes for the eleven TLB-associated parameters in healthy blood donors (BD, n = 55). Table S12: Bivariate analysis of TLB parameters between patients with normal colonoscopy result (CN) and healthy blood donors (BD). Table S13: Main descriptive indexes for the eleven TLB-associated parameters in patients with normal colonoscopy result and without colonoscopy preparation (CNR, n = 55). Table S14: Main descriptive indexes for the eleven TLB-associated parameters in patients with normal colonoscopy result and with colonoscopy preparation (CNN, n = 55). Table S15: Bivariate analysis of TLB parameters of patients with normal colonoscopy result with (CN) and without (CNR) colonoscopy preparation. Table S16: Bivariate analysis of Fluorescence Spectrum parameters (Max and SNR) of patients with normal colonoscopy result with (CN) and without (CNR) colonoscopy preparation. Table S17: Bivariate analysis of albumin and C-reactive protein concentration of patients with normal colonoscopy result with (CN) and without (CNR) colonoscopy preparation.
