# Supplementary material for: Bowel Preparation for Colonoscopy Changes Serum Composition as Detected by Thermal Liquid Biopsy and Fluorescence Spectroscopy
Source: Cancers (Basel). 2023 Mar 24;15(7):1952. doi: 10.3390/cancers15071952 (PMC10093451; doi:10.3390/cancers15071952)
Supplement: Supplementary file 1 [file cancers-15-01952-s001.zip › cancers-2202620-supplementary.pdf]

Supplementary Material

Bowel preparation for colonoscopy changes serum composition as detected by Thermal Liquid Biopsy (TLB) and Fluorescence Spectroscopy. Pre-analytical potential flaw for diagnosis.

Sonia Hermoso-Durán, María José Domper-Arnal, Pilar Roncales Samanes, Sonia Vega, Oscar Sanchez-Gracia, Jorge L. Ojeda, Ángel Lanas, Adrian Velazquez-Campoy and Olga Abian.

Table S1. *Summary of characteristics data of patients from colorectal cancer (CRC) screening program, with fecal immunochemical test positive.*

Age (years; Q2 [Q1-Q3])	60 [56-64]
Sex	60.70 % male<br>39.30 % female
Colonoscopy	48.05 % normal<br>14.79 % non-advanced polyp<br>32.10 % advanced polyp<br>5.06 % CRC
Hemoglobin concentration in fecal immunoassay (ng Hb/mL Buffer; Q2 [Q1-Q3])	381 [200.8-829.5]
Cancer diagnosis	91.63 % no<br>8.37 % yes (from 1984 to 2019)
Mellitus Diabetes diagnosis	84.44 % no<br>15.56 % yes
Autoimmune Disease diagnosis	84.24 % no<br>15.76 % yes
Autoimmune treatment	97.08 % no<br>2.92 % yes

Table S2. *Age distribution for patients from (CRC) screening program according to the sex and to the colonoscopy findings.*

**Sex**	***n* (%)**	**Minimum**	**Q1**	**Q2**	**Average**	**Q3**	**Maximum**
Male	311 (60.51 %)	49.00	56.00	60.00	59.60	63.00	70.00
Female	203 (39.49 %)	49.00	55.50	60.00	60.44	65.50	70.00
Total	514	49.00	56.00	60.00	59.93	64.00	70.00
**Colonoscopy**	***n* (%)**	**Minimum**	**Q1**	**Q2**	**Average**	**Q3**	**Maximum**
Normal	247 (48.05 %)	49.00	55.00	60.00	59.75	64.00	70.00
Pathological	267 (51.95 %)	49.00	56.00	60.00	60.10	64.00	70.00
Non-advanced	76 (28.46 %)	50.00	57.50	61.00	60.60	65.00	70.00
Advanced	165 (61.80 %)	49.00	57.50	60.00	60.11	64.00	70.00
CRC	26 (9.74 %)	51.00	54.00	58.00	58.50	61.75	70.00

Table S3. *Descriptive of sex for patients from (CRC) screening program depending on colonoscopy result.*

Sex	Colonoscopy		*p*-value
	**Normal (*n* = 247)**	**Pathological (*n* = 267)**	
Male (*n* = 311)	117 (47.37 %)	194 (72.66 %)	**< 0.001**
Female (*n* = 203)	130 (52.63 %)	73 (27.34 %)	

Notes: *p*-value were calculated according to Pearson Chi-squared test.

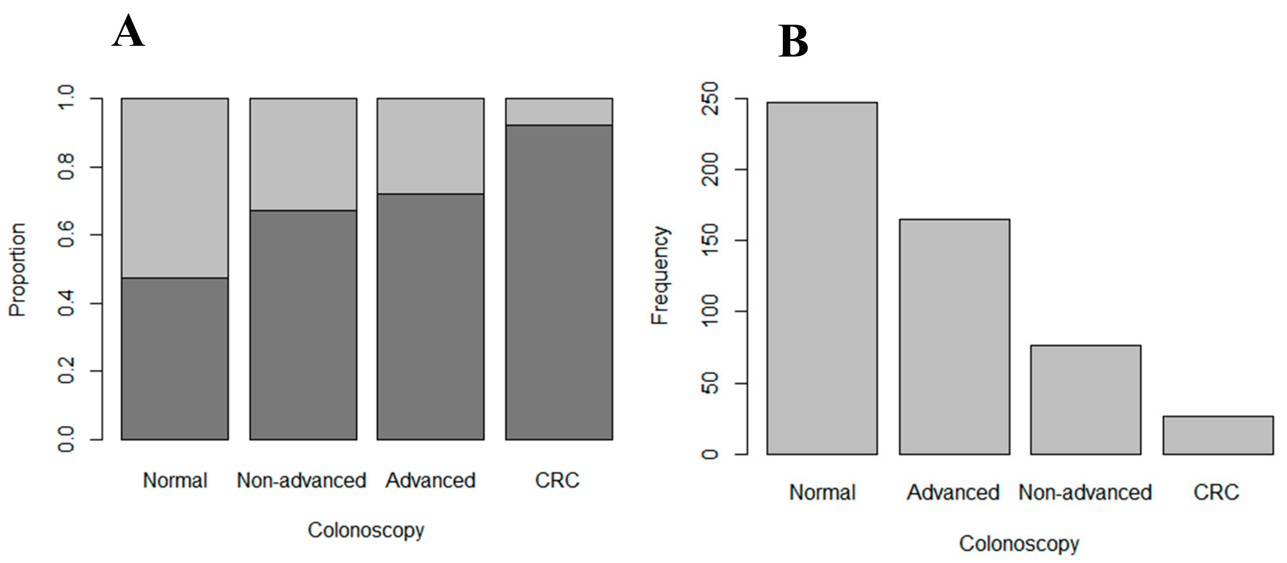

Figure S1: A) *Proportion of males (dark grey) and females (light grey) according to the colonoscopy result, for patients from CRC screening program.* **B)** *Distribution of colonoscopy results for patients from CRC screening program.*

Table S4. *Descriptive of sex for patients from (CRC) screening program depending on each category of colonoscopy result.*

Sex	Colonoscopy			
	**Normal**	**Non-advanced**	**Advanced**	**CRC**
Male (*n* = 311)	117 (47.37 %)	51 (67.11 %)	119 (72.12 %)	24 (92.31 %)
Female (*n* = 203)	130 (52.63 %)	25 (32.89 %)	46 (27.88 %)	2 (7.69 %)
Total (*n* = 514)	247	76	165	26

Table S5. *Age distribution for patients from (CRC) screening program with normal colonoscopy result and without colonoscopy preparation (CNR, n = 55) according to the gender.*

Sex	*n* (%)	Minimum	Q1	Q2	Average	Q3	Maximum
Male	27 (49.10 %)	52.00	58.50	60.00	59.93	62.00	67.00
Female	28 (50.90 %)	51.00	55.50	60.00	60.07	64.25	68.00
Total	55	51.00	57.50	60.00	60.00	62.00	68.00

Table S6. *Age distribution for healthy blood donors (BD, n = 55) according to the sex.*

Sex	*n* (%)	Minimum	Q1	Q2	Average	Q3	Maximum
Male	47 (85.45 %)	50.00	54.00	58.00	57.96	62.00	68.00
Female	8 (14.55 %)	50.00	51.50	57.00	55.75	59.00	62.00
Total	55	50.00	53.50	58.00	57.64	61.50	68.00

Table S7: *Main descriptive indexes for the eleven TLB-associated parameters in patients with normal colonoscopy result (CN, n = 247) and pathological colonoscopy result (CP, n = 267).*

Normal	Minimum	Q1	Q2	Average	Q3	Maximum
Tav	66.68	68.78	69.23	69.32	69.86	62.17
G1	-0.37	0.15	0.28	0.26	0.37	0.64
WP	3.96	7.62	9.99	11.19	13.65	36.34
AUCn2	15.82	36.80	59.32	123.75	94.57	3937.97
APn2	0.23	1.75	4.02	164.43	9.94	20042.62
AUCn3	15.72	20.59	22.45	34.34	25.25	1041.45
APn3	0.19	0.50	0.63	5.48	0.82	538.88
AUCn4	17.97	34.72	45.44	56.56	63.04	394.19
APn4	0.35	1.70	2.59	4.33	4.10	84.11
AUCn5	18.72	23.67	26.98	31.26	33.21	270.45
APn5	0.17	0.63	0.89	1.47	1.49	42.25
**Pathological**	**Minimum**	**Q1**	**Q2**	**Average**	**Q3**	**Maximum**
Tav	66.89	68.64	69.22	69.29	69.80	73.70
G1	-0.34	0.18	0.30	0.27	0.38	0.72
WP	4.34	7.69	10.04	12.66	13.33	437.67
AUCn2	15.91	36.26	54.47	105.62	87.64	5891.25
APn2	0.31	1.68	3.64	100.38	9.17	21190.15
AUCn3	17.40	20.55	22.52	25.79	25.80	118.34
APn3	0.17	0.54	0.63	0.96	0.82	18.67
AUCn4	18.17	33.35	43.52	52.55	57.81	338.08
APn4	0.44	1.56	2.36	3.86	3.64	67.55
AUCn5	17.05	24.01	27.45	32.82	33.26	221.76
APn5	0.15	0.64	0.96	1.92	1.49	68.11

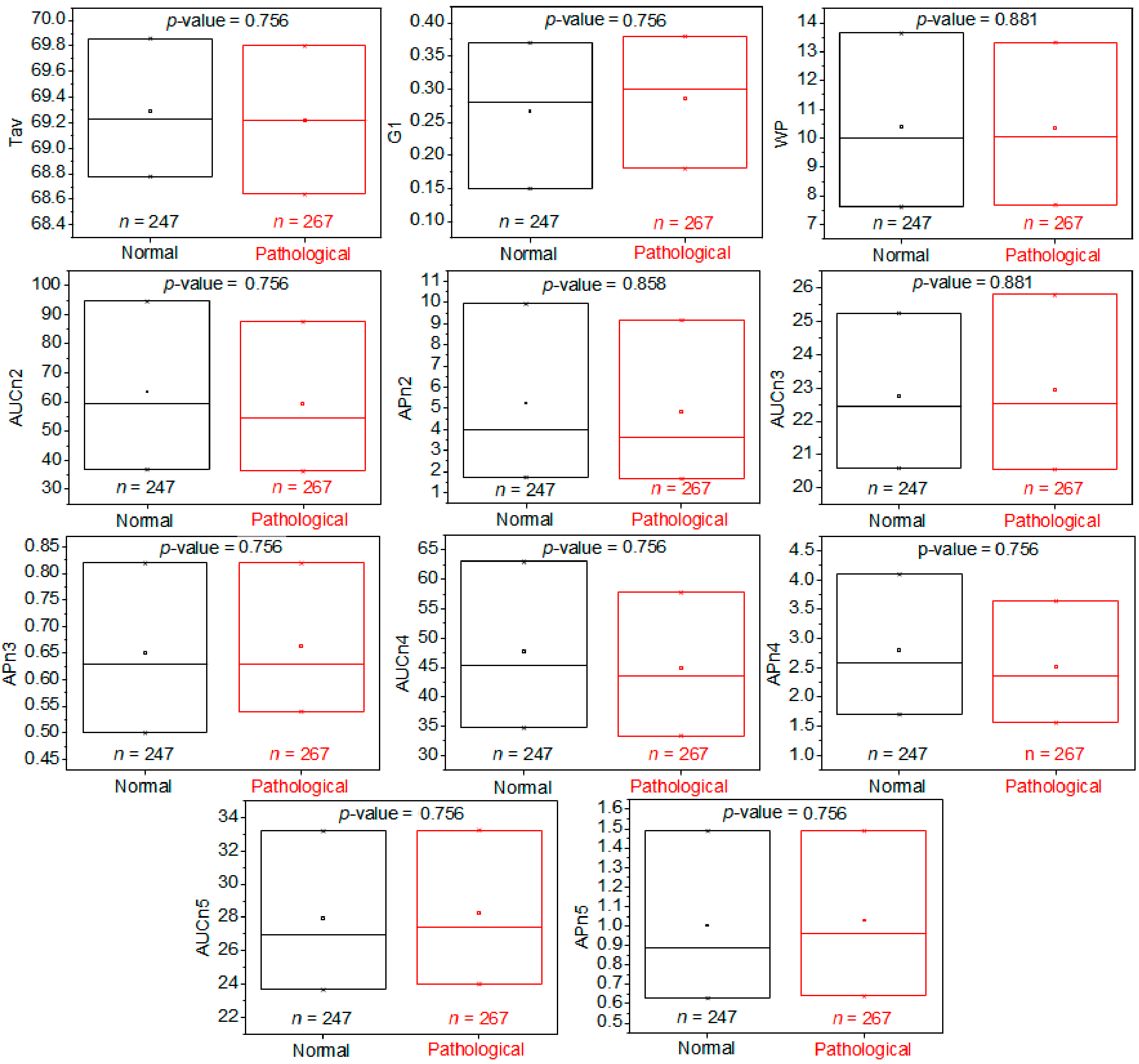

Figure S2: *Box-plot for each individual TLB-parameter illustrating the distribution of the p-values between patients with normal (CN) or pathological colonoscopy (CP) diagnosis.*

Table S8: *Bivariate analysis of TLB parameters of patients with normal colonoscopy result (CN) and patients with pathological colonoscopy result (CP).*

Parameter	Colonoscopy		*p*-value
	**Normal (*n* = 247)**	**Pathological (*n* = 267)**	
Tav	69.23 [68.78;69.86]	69.22 [68.64;69.80]	0.756
G1	0.28 [0.15;0.37]	0.30 [0.18;0.38]	0.756
WP	9.99 [7.62;13.65]	10.04 [7.69;13.33]	0.881
AUCn2	59.32 [36.80;94.57]	54.47 [36.26;87.64]	0.756
APn2	4.02 [1.75;9.94]	3.64 [1.68;9.17]	0.858
AUCn3	22.45 [20.59;25.25]	22.52 [20.55;25.80]	0.881
APn3	0.63 [0.50;0.82]	0.63 [0.54;0.82]	0.756
AUCn4	45.44 [34.72;63.04]	43.52 [33.35;57.81]	0.756
APn4	2.59 [1.70;4.10]	2.36 [1.56;3.64]	0.756
AUCn5	26.98 [23.67;33.21]	27.45 [24.01;33.26]	0.756
APn5	0.89 [0.63;1.49]	0.96 [0.64;1.49]	0.756

Note: median [Q1;Q3]; *p*-values were calculated according to Wilcoxon test for independent samples. P-values are adjusted according to FDR method.

Table S9: *Summary of the application of multivariate Binomial Generalized Linear Model with Logistic Regression (GLM).*

Parameter	*z*-value	*p*-value
Tav	0.558	0.577
G1	0.855	0.393
WP	-0.490	0.624
AUCn2	-0.181	0.856
APn2	0.042	0.966
AUCn3	0.996	0.319
APn3	-1.070	0.285
AUCn4	-1.548	0.122
APn4	1.340	0.180
AUCn5	-1.286	0.198
APn5	1.315	0.188

Note: A cut-off value of 1.96 for *z*-value (which approximately corresponds to a two-sided hypothesis test with a significance level of α = 0.05) indicates whether the corresponding parameters are statistically meaningful in the model. In other words, if the absolute value of the *z*-value is larger than 1.96, then its corresponding GLM coefficient is non-negligible, i.e., not null, and the parameter is important and meaningful for the prediction of the presence/absence of pathological colonoscopy finding.

Table S10: *Contingency table for predict risk of pathological colonoscopy finding.*

Colonoscopy	TLB-score	
	**< 0.50**	**> 0.50**
Normal (*n* = 247)	64	183
Pathological (*n* = 267)	39	228

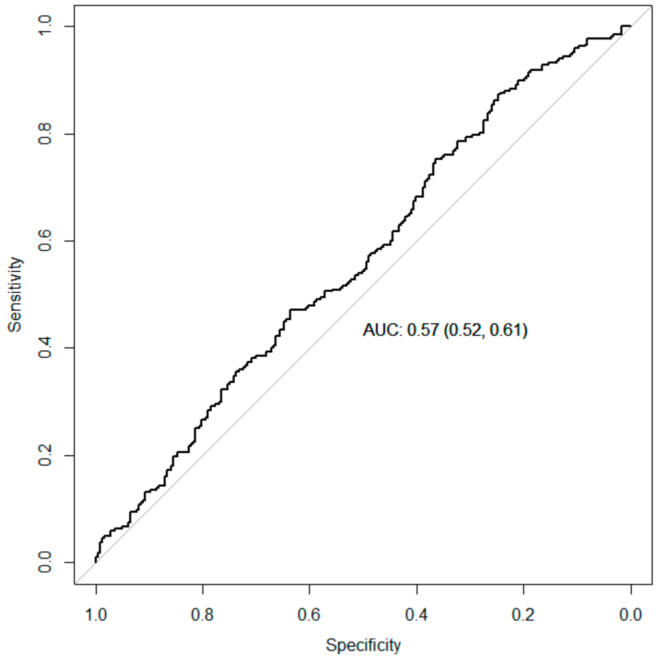

Figure S3: *ROC curve illustrating the statistical performance of the TLB-score and its AUC (95 % CI) for discriminating patients with CN diagnosis from patients with CP diagnosis .*

Table S11: *Main descriptive indexes for the eleven TLB-associated parameters in healthy blood donors (BD, n = 55).*

Parameter	Minimum	Q1	Q2	Average	Q3	Maximum
Tav	67.01	68.02	68.41	68.35	68.81	69.29
G1	0.31	0.37	0.43	0.44	0.49	0.80
WP	5.93	9.35	10.92	11.87	13.95	26.47
AUCn2	15.58	20.62	34.00	41.48	52.73	174.90
APn2	0.19	0.50	1.61	3.40	3.81	46.77
AUCn3	14.62	21.19	23.07	30.06	27.15	214.14
APn3	0.33	0.60	0.74	1.95	0.98	55.89
AUCn4	22.85	32.00	38.94	52.75	54.97	223.13
APn4	0.29	1.36	2.21	5.06	3.49	52.40
AUCn5	23.42	33.36	38.32	47.44	51.58	307.68
APn5	0.50	1.35	2.15	3.56	3.52	43.28

Table S12: *Bivariate analysis of TLB parameters between patients with normal colonoscopy result (C_N) and healthy blood donors (B_D).*

Parameter	Normal Colonoscopy (*n* = 247)	Healthy (*n* = 55)	*p*-value
Tav	69.23 [68.78;69.86]	68.41 [68.02;68.81]	**0.002**
G1	0.28 [0.15;0.37]	0.43 [0.37;0.49]	**0.002**
WP	9.99 [7.62;13.65]	10.92 [9.35;13.95]	**0.044**
AUCn2	59.32 [36.80;94.57]	34.00 [20.62;52.73]	**0.002**
APn2	4.02 [1.75;9.94]	1.61 [0.50;3.81]	**0.002**
AUCn3	22.45 [20.59;25.25]	23.07 [21.19;27.15]	0.289
APn3	0.63 [0.50;0.82]	0.74 [0.60;0.98]	**0.002**
AUCn4	45.44 [34.72;63.04]	38.94 [32.00;54.97]	0.084
APn4	2.59 [1.70;4.10]	2.22 [1.36;3.49]	0.257
AUCn5	26.98 [23.67;33.21]	38.32 [33.36;51.58]	**0.002**
APn5	0.89 [0.63;1.49]	2.15 [1.35;3.52]	**0.002**

Note: median [Q1;Q3]; *p*-values were calculated according to Wilcoxon test for independent samples (normal approximation). P-values are adjusted according to FDR method.

Table S13: *Main descriptive indexes for the eleven TLB-associated parameters in patients with normal colonoscopy result and without colonoscopy preparation (C_{NR}, n = 55).*

Parameter	Minimum	Q1	Q2	Average	Q3	Maximum
Tav	66.63	67.83	68.25	68.47	68.95	71.10
G1	0.01	0.24	0.38	0.37	0.48	0.69
WP	5.74	7.02	7.93	9.23	10.21	10.11
AUCn2	14.98	21.69	34.26	69.81	67.04	1115.48
APn2	0.30	0.73	1.98	25.81	6.01	1074.47
AUCn3	17.90	19.64	21.35	24.40	25.79	110.19
APn3	0.35	0.57	0.70	0.95	0.92	10.41
AUCn4	18.30	30.04	36.28	63.32	47.52	1038.33
APn4	0.38	1.55	2.21	10.00	3.49	364.06
AUCn5	18.17	27.61	34.60	36.78	40.26	135.04
APn5	0.11	1.08	1.78	2.37	2.70	20.93

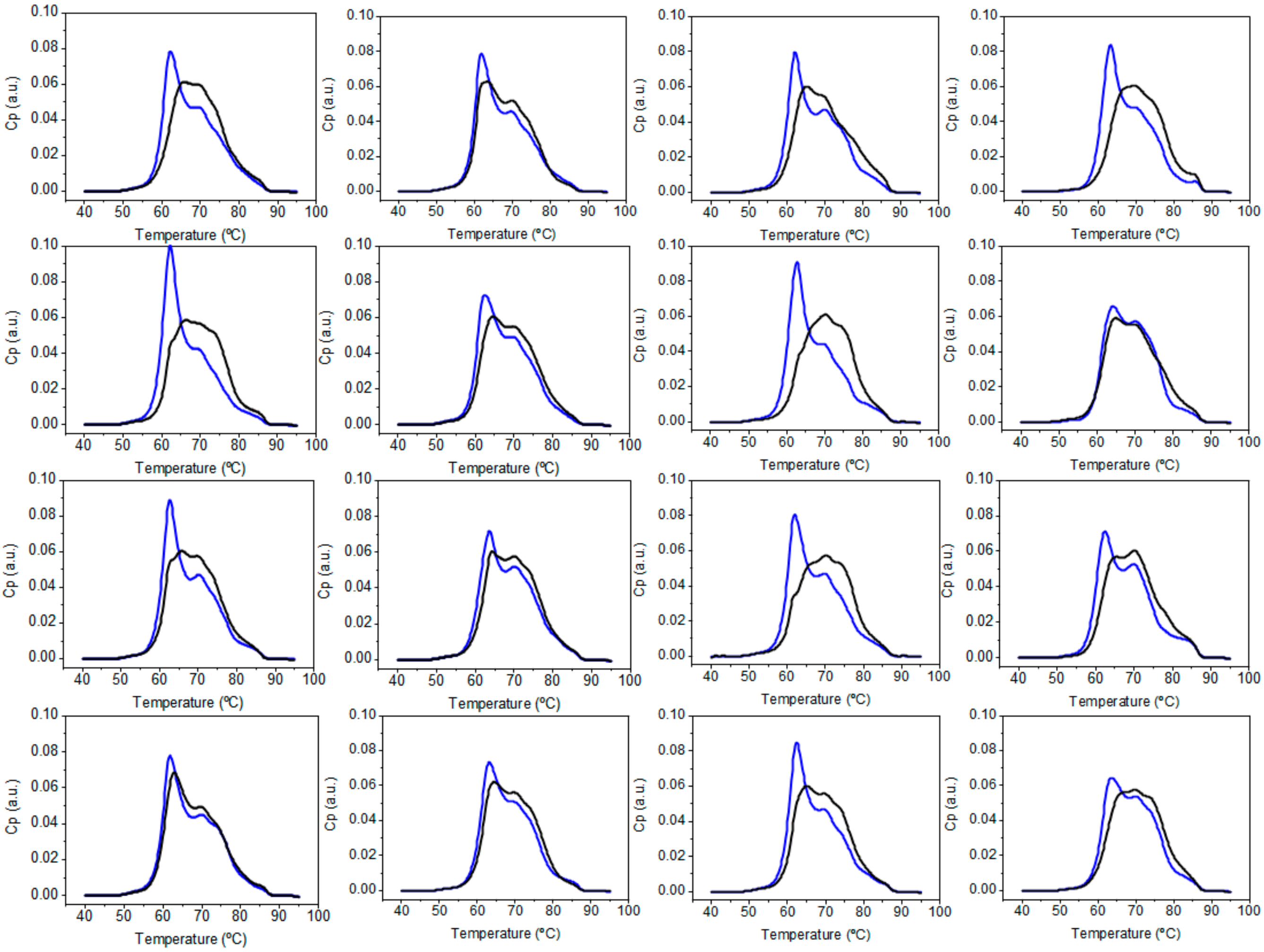

Figure S4: *Comparison of individual examples of 16 patient thermograms with normal colonoscopy diagnosis. Samples were drawn in two different conditions: under colonoscopy preparation (CN, black) and under standard analytical conditions (CNR, blue).*

Table S14: *Main descriptive indexes for the eleven TLB-associated parameters in patients with normal colonoscopy result and with colonoscopy preparation (CN_N, n = 55).*

Parameter	Minimum	Q1	Q2	Average	Q3	Maximum
Tav	67.67	68.81	69.17	69.31	69.86	70.96
G1	0.001	0.15	0.27	0.26	0.34	0.57
WP	4.31	7.11	9.11	10.04	12.38	21.20
AUCn2	21.56	40.86	62.85	111.13	95.21	1079.64
APn2	0.23	1.73	4.45	28.15	10.34	750.19
AUCn3	18.58	20.16	21.48	42.08	24.52	1041.45
APn3	0.25	0.44	0.57	10.46	0.79	538.88
AUCn4	24.26	36.25	48.12	58.96	62.11	393.11
APn4	0.79	1.80	2.65	4.53	3.68	70.84
AUCn5	19.14	23.27	26.43	28.98	31.44	57.11
APn5	0.17	0.53	0.88	1.11	1.43	4.20

Table S15: *Bivariate analysis of TLB parameters of patients with normal colonoscopy result with (CN) and without (CNR) colonoscopy preparation.*

Parameter	Preparation		*p*-value
	**No (*n* = 55)**	**Yes (*n* = 55)**	
Tav	68.47 (0.87)	69.31 (0.80)	**0.002**
G1	0.37 (0.15)	0.26 (0.12)	**0.002**
WP	7.93 [7.02;10.21]	9.11 [7.11;12.38]	0.524
AUCn2	34.26 [21.69;67.04]	62.85 [40.86;95.21]	**0.002**
APn2	1.98 [0.73;6.01]	4.45 [1.73;10.34]	**0.005**
AUCn3	21.35 [19.64;25.79]	21.48 [20.16;24.52]	0.575
APn3	0.70 [0.57;0.92]	0.57 [0.44;0.79]	**0.015**
AUCn4	36.28 [30.04;47.52]	48.12 [36.25;62.11]	**0.002**
APn4	2.21 [1.55;3.49]	2.65 [1.80;3.68]	0.072
AUCn5	34.60 [27.61;40.26]	26.43 [23.27;31.44]	**0.002**
APn5	1.78 [1.08;2.70]	0.88 [0.53;1.43]	**0.002**

Note: average (standard deviation) and median [Q1;Q3] are provided depending on the normality character of the parameter distribution; *p*-values were calculated according to T-Student or Wilcoxon test , both for dependent samples, depending on the normality character of the parameter distribution. P-values are adjusted according to FDR method.

Data Analysis. We have developed a phenomenological model in which the complex thermogram was deconvoluted in several individual transitions or components, modelling each individual transition by the logistic peak or Hubbert function:

$$C_P(T) = C_{P,0} + \frac{4A exp\left(-\frac{T-T_C}{w}\right)}{\left(1+exp\left(-\frac{T-T_C}{w}\right)\right)^2} \qquad \text{(S1)}$$

where A is the height of the transition (equivalent to the maximal unfolding heat capacity $C_{P,max}$), T_c is the center of the peak (equivalent to the mid-transition temperature T_m), and w is the width of the peak ($C_P(T_c \pm w) = 0.8A$ and $C_P(T_c \pm 2w) = 0.4A$). The offset parameter $C_{P,0}$ (found to be always very close to 0 in the experimental data analysis), was included as an adjustable parameter to counterbalance errors from baseline correction of the thermogram.

From our experience, a minimum set of six individual curves was necessary for reproducing the serum thermograms:

$$C_P(T) = C_{P,0} + \sum_{i=1}^{6} \frac{4A_i exp\left(-\frac{T-T_{C,i}}{w_i}\right)}{\left(1+exp\left(-\frac{T-T_{C,i}}{w_i}\right)\right)^2} \qquad \text{(S2)}$$

Therefore, for any given serum thermogram eighteen parameters (A_i, $T_{c,i}$ and w_i, for each of the six individual transitions) were obtained after data analysis.

The area under the curve, AUC, and the average temperature, T_{av}, were defined according to the following expressions:

$$AUC = \sum_j C_P(T_j)$$
$$T_{av} = \frac{\sum_j C_P(T_j) T_j}{\sum_j C_P(T_j)} \qquad \text{(S3)}$$

where j runs over the entire range of experimental points in the thermogram.
From T_{av}, which is the first raw moment (μ_1) of the thermogram considered as a distribution, two other moments about T_{av}, m_2 and m_3, were defined as:

$$m_k = \frac{\sum_j C_P(T_j)(T_j - T_{av})^k}{\sum_j C_P(T_j)}$$
$$G_1 = \frac{m_3}{m_2^{3/2}} \qquad \text{(S4)}$$

where G_1 is the skewness of the thermogram (i.e., the asymmetry of the thermogram with respect to T_{av}).
The parameter WP, the area of the width-polygon, is the area of the 6-side polygon constructed with the widths of the six individual components:

$$WP = \sum_{s=1}^{6} \frac{\sqrt{3}}{4} w_s w_{s+1} \qquad \text{(S5)}$$

where $w_7 = w_1$.
The height-normalized area under the curve, $AUC_{n,i}$ was defined as:

$$AUC_{n,i} = \sum_j \frac{C_P(T_j)}{A_i} \qquad \text{(S6)}$$

where the normalization is done with respect to A_i (i = 2-5).
And the area of the height-normalized polygon, $AP_{n,i}$, is the area of the 6-side polygon constructed with the heights of the six individual components normalized by A_i (i = 2-5):

$$AP_{n,i} = \sum_{s=1}^{6} \frac{\sqrt{3}}{4} \frac{A_s A_{s+1}}{A_i^2} \qquad \text{(S7)}$$

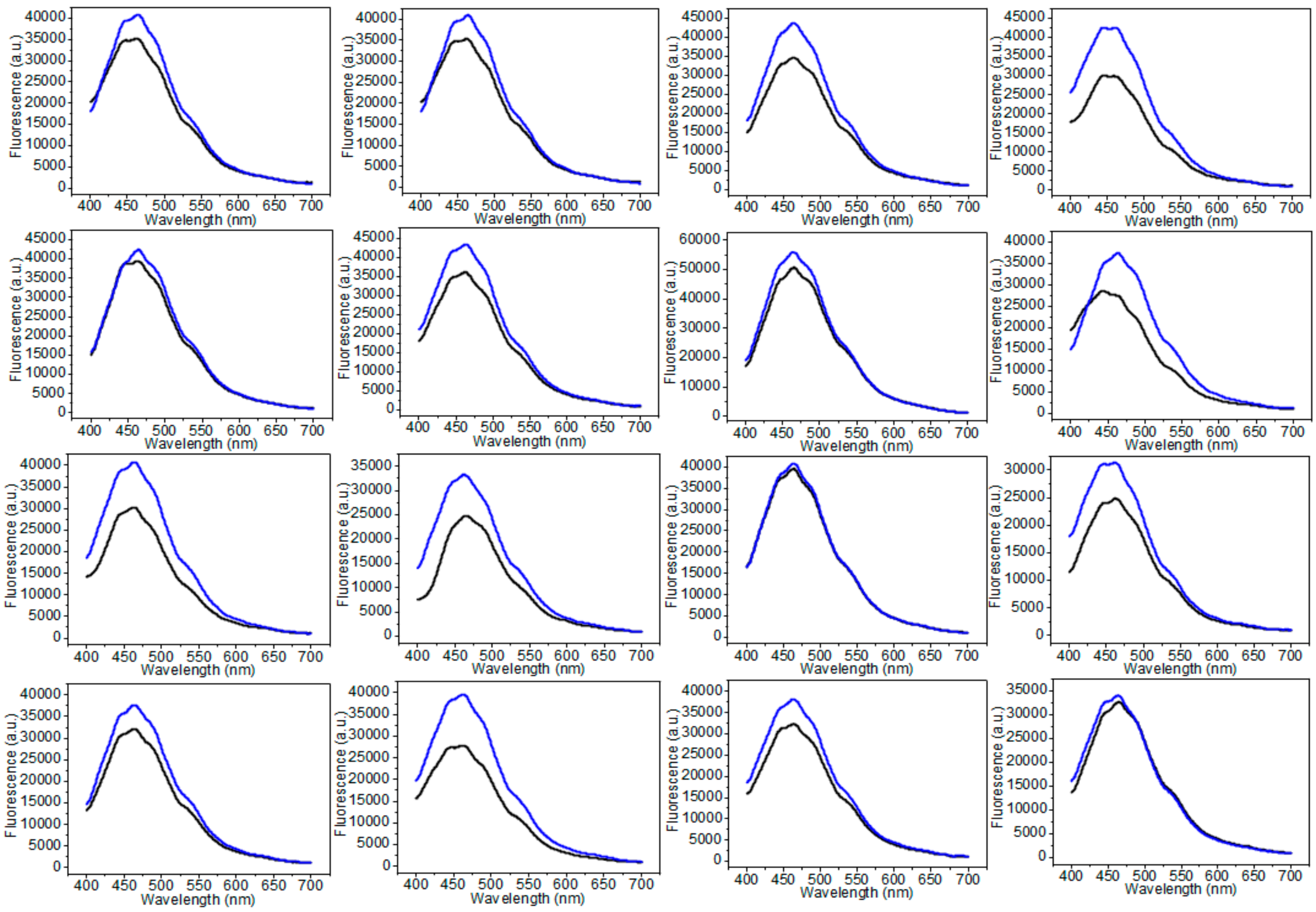

Figure S5: *Comparison of individual examples of 16 patient fluorescence spectra with normal colonoscopy diagnosis. Samples were drawn in two different conditions: under colonoscopy preparation (CN, black) and under standard pre-analytical conditions (CNR, blue).*

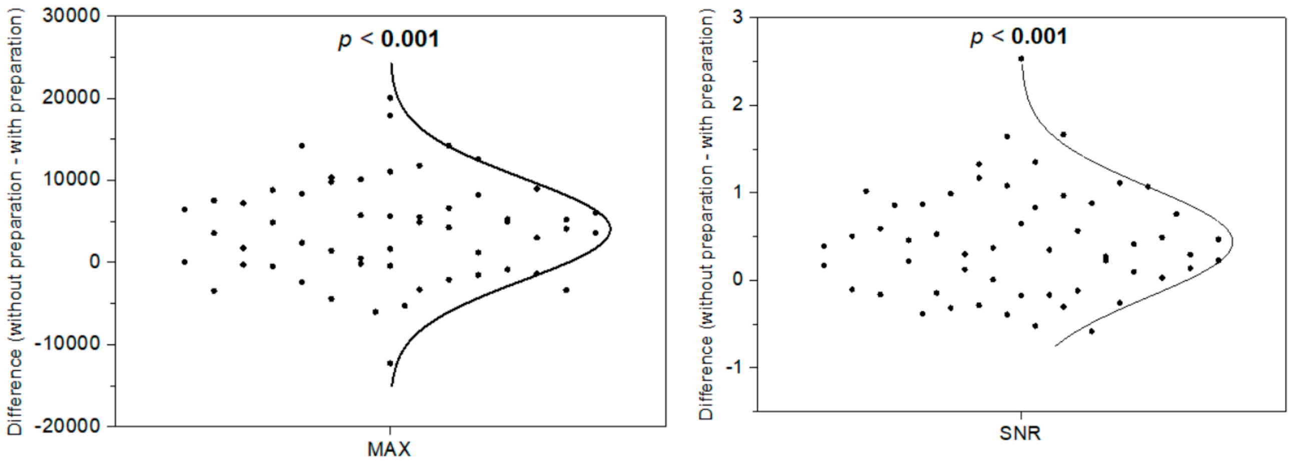

Figure S6: *Plot and distribution of the difference in MAX and SNR between samples without preparation and samples with preparation.*

Note: *p*-values were calculated according to T-Student test for dependent samples.

Table S16: *Bivariate analysis of Fluorescence Spectrum parameters (Max and SNR) of patients with normal colonoscopy result with (CN) and without (CNR) colonoscopy preparation.*

Parameter	Preparation		*p*-value
	No (*n* = 55)	Yes (*n* = 55)	
Max	39346.13 (7462.28)	35305.68 (7393.88)	**< 0.001**
SNR	7.67 (0.46)	7.238 (0.64)	**< 0.001**

Note: average (standard deviation); *p*-values were calculated according to T-Student test for dependent samples.

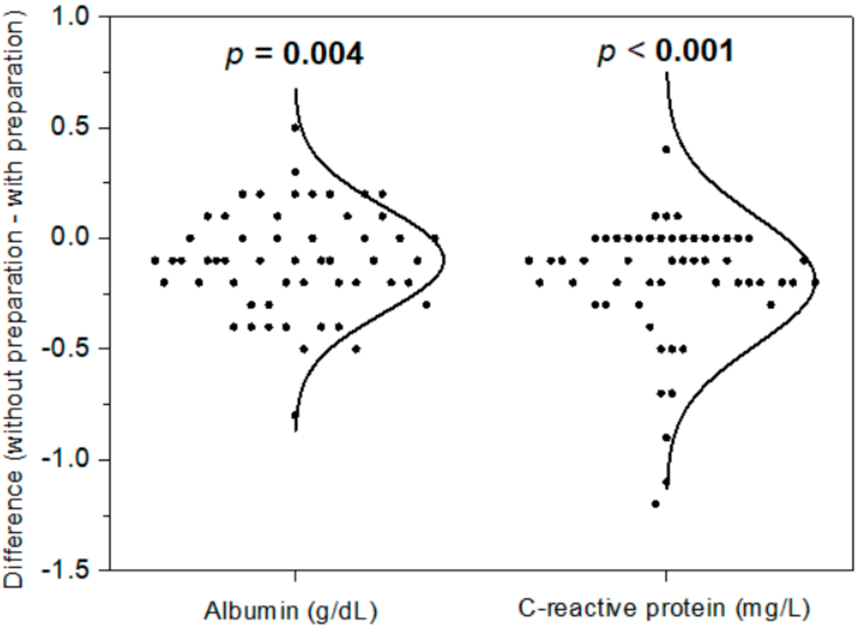

Figure S7: *Plot and distribution of the difference in albumin concentration and C-reactive protein concentration between samples without preparation and samples with preparation.*
Note: *p*-values were calculated according to T-Student or Wilcoxon test, both for dependent samples , depending on the normality character of the parameter distribution.

Table S17: *Bivariate analysis of albumin and C-reactive protein concentration of patients with normal colonoscopy result with (CN) and without (CNR) colonoscopy preparation.*

Parameter	Preparation		*p*-value
	No (*n* = 55)	Yes (*n* = 55)	
Albumin (g/dL)	4.48 (0.29)	4.58 (0.27)	**0.004**
C-Reactive Protein (mg/L)	0.20 [0.10;0.30]	0.30 [0.20;0.50]	**< 0.001**

Note: average (standard deviation) and median [Q1; Q3] are provided depending on the normality character of the parameter distribution; *p*-values were calculated according to T-Student or Wilcoxon test , both for dependent samples , depending on the normality character of the parameter distribution.
